# Label-Free Morpho-Molecular Imaging for Studying the Differential Interaction of Black Phosphorus with Tumor Cells

**DOI:** 10.3390/nano12121994

**Published:** 2022-06-10

**Authors:** Valentina Mussi, Ines Fasolino, Debadrita Paria, Sara De Simone, Maria Caporali, Manuel Serrano-Ruiz, Luigi Ambrosio, Ishan Barman, Maria Grazia Raucci, Annalisa Convertino

**Affiliations:** 1 Institute for Microelectronics and Microsystems, National Research Council, 00133 Rome, Italy; valentina.mussi@cnr.it (V.M.); sara.desimone@artov.imm.cnr.it (S.D.S.); 2 Institute of Polymers, Composites and Biomaterials, National Research Council, 80125 Naples, Italy; ines.fasolino@gmail.com (I.F.); luigi.ambrosio@cnr.it (L.A.); 3 Department of Mechanical Engineering, Johns Hopkins University, Baltimore, MD 21218, USA; dparia1@jhu.edu; 4 Institute for the Chemistry of Organometallic Compounds, National Research Council, 50019 Sesto Fiorentino, Italy; maria.caporali@iccom.cnr.it (M.C.); manuel.serrano@iccom.cnr.it (M.S.-R.); 5 Department of Oncology, Johns Hopkins University School of Medicine, Baltimore, MD 21218, USA; 6 Department of Radiology & Radiological Science, Johns Hopkins University School of Medicine, Baltimore, MD 21218, USA

**Keywords:** black phosphorus, optical diffraction tomography, Raman mapping, cellular internalization

## Abstract

Black phosphorus nanosheets (2D BP) are emerging as very promising, highly selective chemotherapeutic agents due to their fast degradation in the intracellular matrix of cancer cells. Here, optical diffraction tomography (ODT) and Raman spectroscopy were exploited as a powerful label-free approach to achieve integrated insights into the processes accompanying the administration of exfoliated 2D BP flakes in human prostatic adenocarcinoma and normal human prostate epithelial cells. Our ODT experiments provided unambiguous visualization of the 2D BP internalization in cancer cells and the morphological modifications of those cells in the apoptotic phase. The cellular internalization and damaging occurred, respectively, 18 h and 36–48 h after the 2D BP administration. Changes in the chemical properties of the internalized 2D BP flakes were monitored by Raman spectroscopy. Interestingly, a fast oxidation process of the 2D BP flakes was activated in the intracellular matrix of the cancer cells after 24 h of incubation. This was in sharp contrast to the low 2D BP uptake and minimal chemical changes observed in the normal cells. Along with the understanding of the 2D BP fate in the cancer cells, the proposed label-free morpho-molecular approach offers a powerful, rapid tool to study the pharmacokinetic properties of engineered nanomaterials in preclinical research.

## 1. Introduction

Our continuously improving ability to design nanomaterials that can selectively probe and destroy cells in the human body has opened the door to the development of promising approaches for early disease diagnosis and personalized therapies [1,2]. Several nanostructured materials have been conceptualized with diverse physicochemical properties and endowed with different biological and medical capabilities. For example, metal nanoparticles (NPs) have been demonstrated as effective photothermal agents for in vivo imaging and destruction of cancer cells [3,4,5] as well as targeted drug nanocarriers [6,7]. In vitro, other nanomaterials such as silicon nanowires (SiNWs) have shown tremendous promise in recording extracellular and intracellular bioelectrical signals [8,9,10,11], destroying tumor cells [12], detecting very low concentrations of different biomolecules [13,14,15,16], and offering mechanotyping possibilities [17,18].

Recently, the family of nanomaterials suitable for biomedical applications has expanded to include two-dimensional (2D) materials, notably, graphene and Xenes (e.g., borophene, silicene, germanene, stanene, phosphorene, arsenene, antimonene, and tellurene). The 2D materials have been proposed for disease diagnosis using different imaging modalities [19,20,21,22] and for photothermal therapy [23,24]. Interestingly, black phosphorous (2D BP) flakes have shown early promise in the selective destruction of cancer cells while remaining benign towards healthy cells within a certain dosage range. This exceptional feature of 2D BP has been demonstrated for several cancer cell lines, including human cervical cancer cells, human alveolar adenocarcinoma, human lung carcinoma, human hepatoma, and prostate cancer cells [25,26,27,28,29]. On the other hand, healthy cells, such as human bone marrow stem cells, human adult osteoblasts, human fibroblast, and prostate epithelial cells, remain unaffected by exposure to 2D BP [27,28,29].

While most of the characterization efforts are mainly focused on the investigation of cancer cells’ fate after the administration of or exposure to nanomaterials intended for diagnostic and therapeutic use, there is a lack of explorative methodologies that can directly detect and characterize the nanomaterials in the intracellular environment. Characterization of the uptake, translocation, accumulation, and physicochemical changes of nanomaterials as well as well as the detection of the morphological changes in the treated cells are crucial for a better design of nanomaterial sensing and pharmacological therapeutic properties. The selective therapeutic action of 2D BP represents a particularly complex and poorly understood process, a better understanding of which demands detection of the 2D BP internalization and degradation, considered the cause of cellular damage and killing. Indeed, evidence from recent studies [30,31,32] indicates that the 2D BP degradation proceeds spontaneously in the presence of water and oxidative stress, providing phosphate anions as the major degradation products [32]. Thus, the strong intracellular oxidative stress and accelerated energy metabolism affecting the cancer cells cause a fast oxidation process of internalized 2D BP flakes and the production of phosphate anions that, in turn, stimulate significantly higher reactive oxygen species’ (ROS) level in cancer cells in respect to the normal counterparts [27,28]. The increase in the ROS level in tumor cells leads to significant changes in the cytoskeleton, cell cycle arrest, irreparable DNA damage, and apoptosis. The prevalent approach to study this phenomenon is based on the use of immunofluorescence analysis [26,27,28,29] that allows cellular imaging and gives only information about cell behavior after cell–material interaction without providing any information on the 2D BP fate. In addition, this technique suffers from various drawbacks, such as the perturbation of the intrinsic intracellular matrix by the introduction of exogenous fluorescence tags and the difficulty of long-term imaging due to photo-bleaching.

Here, we performed a label-free investigation of the internalization and oxidation processes of exfoliated 2D BP flakes in human prostate adenocarcinoma cells (PC-3) by using optical diffraction tomography (ODT) and Raman spectroscopy. The ODT technique, being label-free and non-invasive, allows long- time monitoring of biochemical and morphological alterations, along with tracking endocytic activity introduced by the presence of the 2D BP flakes. Specifically, ODT [33,34,35] has the ability to image live cells continuously for a few days by measuring the three-dimensional (3D) distribution of their refractive index (RI), which is an intrinsic optical property of the cell, thus not requiring the addition of any contrast agent. The diffracted optical field is detected using Mach–Zehnder interferometry, and the 2D images acquired at various angles are reconstructed to provide a 3D RI image. In our experiments, the local change in RI due to the presence of the 2D BP flakes was distinctly visible in the holographic image, allowing direct and unimpeded observation of internalization of the flakes, their localization inside the cell, and the change in cellular morphology because of endocytosis. On the other hand, Raman spectroscopy can directly probe the changes in the chemical properties of the 2D BP flakes occurring during those processes. In fact, a recent work demonstrated that the degradation process and etching dynamics of 2D BP in ambient conditions can be observed by Raman spectroscopy [36]. It was found that the variation in the measured Raman intensity and full width half maximum (FWHM) for A^1^_g_, B_2g,_ and A^2^_g_ vibrational modes of the 2D BP flakes followed the evolution of the oxidation mechanism and etching dynamics until the complete degradation of the exfoliated 2D BP flakes [36]. In our study, the processes, accompanying the interaction between the PC-3 cells and 2D BP flakes, were observed over an incubation time of the cells with 2D BP ranging from 10 h to 72 h and compared to the human normal prostate epithelial (PNT-2) cells, adopted as the healthy model. The experiments were performed by a fixed dosage of 5 μg/mL of the 2D BP flakes, whose cytotoxic effects on the cancer cells and biocompatible behavior for the healthy ones were observed through confocal microscopy after 72 h of exposure. Our ODT and Raman investigations revealed a fast internalization of the 2D BP flakes in the tumor cells and their successive oxidation inside the tumor intracellular matrix within 24 h after incubation, as well as the transition of the PC-3 cells into the apoptotic state within 48 h after the administration.

Overall, our study showed how ODT and Raman spectroscopy together provide a powerful, label-free approach to visualize and investigate the 2D BP flakes internalized in the cells, allowing us to reveal hitherto inaccessible information on 2D BP inside the intracellular environment. By building our knowledge of the differential interactions of the 2D BP flakes with cells from the bottom up, these combined methods form the foundation of a fresh, innovative approach informing the efficient and safe use of 2D BP as a therapeutic agent. More generally, the internalization of engineered nanomaterials into target cells and the related physicochemical processes, activated by the interaction of these synthetic materials with membrane surfaces, endosomal compartments, organelles, and cytoplasm, are key to developing promising therapeutic and/or diagnostic applications in nanomedicine. We expect that the synergistic combination of ODT and Raman microscopy will provide a broadly applicable tool to generate a better understanding of these mechanisms in diverse systems.

## 2. Materials and Methods

### 2.1. Synthesis of 2D BP Flakes

The 2D BP flakes were obtained by liquid phase exfoliation of bulk BP prepared according to the literature [37,38,39]. The liquid phase exfoliation was performed by suspending BP microcrystals (5.0 μg, 0.161 mmol) in 5.0 mL of dry dimethylsulfoxide (DMSO) (Sigma-Aldrich, St. Louis, MO, USA) in a quartz vial. Then, deoxygenated water (3.0–5.0 μL, 0.167–0.278 mmol) was added to obtain a molar ratio P/H_2_O of 1–0.6. After deoxygenating the suspension by vacuum–nitrogen cycles, the vial was sealed and kept in an ultrasonic bath (37 MHz) for 5 days at 30 °C in the darkness. Degassed ethanol (10.0 mL) was added, the suspension was centrifuged for 30 min at 9500 rpm, the supernatant was removed, and the treatment was repeated twice. The resulting dark gray powder was re-suspended by sonication (approximately 1 min) in deionized water (1 mg/mL) and 1% of DMSO; the suspension was diluted in distilled water to obtain a solution at 0.1% of DMSO that is not toxic to the cells. Later, the desired amount of this suspension was drop-casted on the well plate and left in ambient conditions for the solvent to evaporate. The morphology, purity, and crystallinity of exfoliated black phosphorus were earlier reported [39].

### 2.2. Study of 2D BP Morphology

SEM observations were carried out at Ce.ME CNR (Sesto Fiorentino, Italy) using a Dual Beam, TESCAN GAIA3 FIB/SEM (Brno, Czech Republic) ultrahigh-resolution field emission microscope at 5 KeV voltage. A few drops of 2D BP suspended in acetone were placed on the copper/carbon grid, dried under a stream of nitrogen, and measured.

### 2.3. Cell Culture

The experiments were performed using human Caucasian prostate adenocarcinoma (PC-3) and human normal prostate epithelium immortalized with SV40 (PNT-2) cell lines. Cell lines and materials were purchased from Sigma-Aldrich (St. Louis, MO, USA). Cells were cultured in a 75 cm^2^ cell culture flask in RPMI 1640 and Dulbecco’s modified eagle’s medium (DMEM), supplemented with 10% fetal bovine serum (FBS), antibiotic solution (streptomycin 100 µg/mL and penicillin 100U/mL) and 2 mM l-glutamine. The PC-3 cells, at passages 11–15, and PNT-2 cells, at passages 9–11, were used for all the experimental tests and were grown at 37 °C in an incubator containing a humidified atmosphere with 5% CO_2_ and 95% air.

### 2.4. Cell Proliferation

PC-3 and PNT-2 proliferation was tested by Alamar Blue assay after 72 h of seeding onto 2D BP flakes. Optical density was measured with a UV-Vis spectrophotometer (Victor X3 Multilabel Plate Reader, Perkin Elmer, Waltham, MA, USA) at wavelengths of 570 and 600 nm after 4 h of incubation at 37 °C, which is the time required for the cells to convert resazurin to resorufin. The optical density detected was directly proportional to metabolic activity of live cells.

### 2.5. Cell Morphology

To verify the effect of 2D BP flakes on cell morphological changes after oxidative stress induction, immunofluorescence analysis was carried out. To evaluate the effect of oxidative stress on PNT-2 and PC-3 morphology after 72 h of cell–material interaction, qualitative analysis was performed using Rhodamine phalloidin staining and analyzed in a confocal microscope (Leica TCS sp8 confocal microscope, Wetzlar, Germany) after cell fixation in 4% formaldehyde overnight at 4 °C.

### 2.6. Cell Permeability and Apoptosis Detection

The penetration of the 2D BP flakes inside the PC-3 cells and apoptosis induction were verified through Annexin V-AF647/PI Cell Apoptosis Detection kit. This assay allows distinguishing cells at different death stages because of Annexin V’s ability to bind phosphatidylserine (PS), one of the earliest indicators of apoptosis, in a calcium-dependent manner when PS is on the outer surface of the plasma membrane. Meanwhile, propidium iodide (PI) is a common DNA dye that is not permeable to the cell membrane; but, due to the loss of integrity of the membrane, PI can enter late in apoptotic cells to stain DNA. Hence, PC-3 and PNT-2 cells were grown in the presence and in the absence of 2D BP and processed as previously described for cell morphology analysis. Afterwards, at the fixation stage, the cells were stained with the components of the Annexin V-AF647/PI kit (Elabscience^®^, Wuhan, China) and analyzed using a confocal microscope (Leica TCS sp8).

### 2.7. Optical Diffraction Tomography and Statistical Analysis

The PC-3 cells were seeded on a glass-bottom Petri dish for the ODT imaging (HT-1H, Tomocube Inc., Republic of Korea) using a 60× N.A–1.2) water immersion objective. The 2D BP flakes were added to the cell culture after 24 h of seeding the cells on the Petri dish to ensure proper attachment and spreading of the cells on the dish. Before the ODT imaging, the cells were washed with phosphate-buffered saline (PBS) to remove the 2D BP flakes in the solution and a fresh medium was reintroduced in the Petri dish. An in situ incubator attached to the imaging setup was used for keeping the cells alive during the experiment. Throughout the duration of the experiment a temperature of 37 °C, a CO_2_ level of 5%, and a humidified atmosphere were maintained to preserve the pristine condition of the cell. A digital micromirror device along with a 532 nm laser enabled tomographic scanning of the live cells [40]. Fourier diffraction theorem was used to reconstruct a 3D RI image of the cells from the obtained interferograms [41]. The 3D maps of live cells and their 2D maximum intensity projections (MIP) were generated, visualized, and analyzed in TomoStudio software (Tomocube Inc, Daejeon, Korea). For the morphological analysis of the cells, CellProfiler^TM^ (v3.1.9) software was used to segment the MIP images to identify and isolate a single cell. The area and aspect ratio (major/minor axis lengths) were calculated in the CellProfiler^TM^ by considering non-zero pixels in the segmented mask [42]. A statistical significance of differences in area and the aspect ratio between the different time points were calculated using a one-sided Student’s *t*-test with significance level considered as 0.05. Cohen’s d, d = (μ_1_ − μ_2_)/s (μ_1_, μ_2_: means of the two time points used for comparison; s: pooled standard deviation), was used for effect size calculation.

### 2.8. Raman Microscopy

The samples were prepared by seeding PC-3 and PNT-2 cells onto the 2D BP flakes for different time intervals of 10 h, 24 h, 48 h, and 72 h. Afterwards, fixation of the samples for Raman measurements was performed using 4% formaldehyde overnight at 4 °C, followed by washing for three times and storing in PBS 1X until Raman analysis. Raman maps of the treated and untreated cells were then acquired with a DXR2xi Thermo Fisher Scientific (Waltham, MA, USA) Raman imaging microscope by exciting the samples at 532 nm with 2 mW laser power and a 50× objective. Each point spectrum resulted from 30 accumulations of 0.1 s acquisitions, and the maps were collected over a square area with a fixed step size of 1 μm. Each map contained several cells and many 2D BP flakes, localized inside, near, and far from the cells depending on the cell status, type, and time interval from the seeding. For every map, the 2D BP aggregates were visually and spectrally identified, and the corresponding point spectra were individually analyzed by means of a Lorentzian fitting procedure to extract the FWHM of the three peaks ascribed to 2D BP. For each time interval, the fitting results were used to build separate histograms for 2D BP decorating the cells or far away from them.

## 3. Results and Discussion

### 3.1. Immunofluorescence Investigation on PC-3 and PNT-2 Cells Seeded on 2D BP Flakes

**Figure 1:**
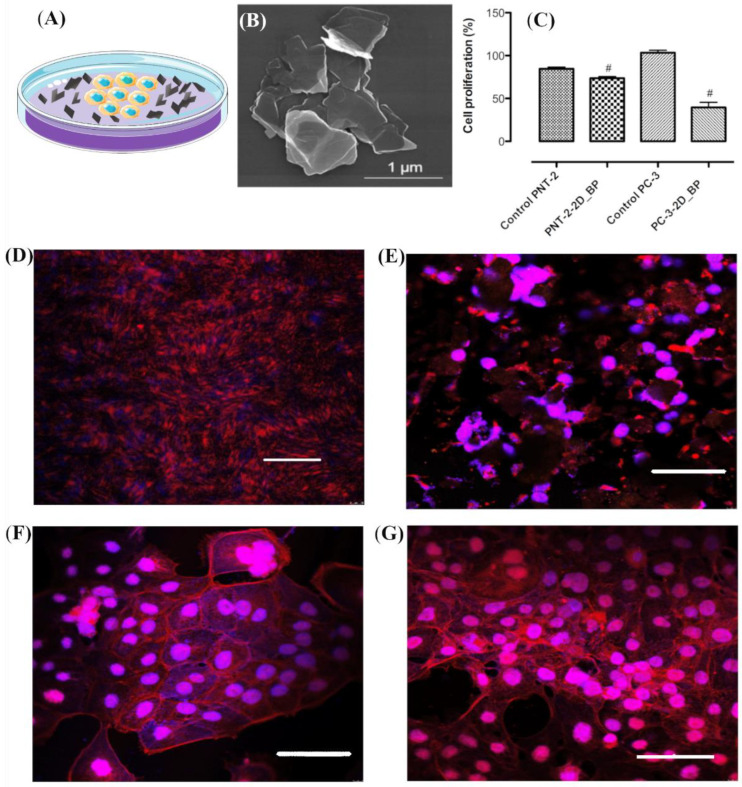
Schematic representation of the cells seeded on a Petri dish with 2D BP flakes (5 μg/mL) (**A**). SEM image of 2D BP flakes (**B**). Cell proliferation analysis on PC-3 and PNT-2 cultured with and without 2D BP (control). Alamar Blue assay shows that 2D BP did not exert cytotoxic effects on PNT-2 after 72 h of exposure. By contrast, 2D BP induced a significant decrease in PC-3 (cancer cells) proliferation after 72 h of exposure compared to control (*#* indicates statistically significant differences at the threshold value of *p* < 0.0001). Results are mean ± SEM of at least three independent experiments (**C**). Immunofluorescence analysis, PC-3 and PNT-2 grown for 72 h in absence (**D**,**F**) and presence of 2D BP flakes ((**E**,**G**), respectively); scale bar: 200 μm.

PC-3 and PNT-2 cells, roughly 1.5 × 10^4^ cells, were seeded on Petri dishes with exfoliated 2D BP flakes at the bottom of the plate, as shown in Figure 1A. The exfoliated 2D BP flakes, whose SEM image is in Figure 1B, were characterized by a sheet-like morphology with lateral dimensions ranging from ca. 300 nm to 1.2 μm, as previously discussed in detail [39]. In addition, the atomic force microscopy (AFM) analysis, already reported in Ref. [39], confirmed the nanosheets to be homogeneous on a large scale with heights ranging from a few nanometers to 10 nanometers. The 2D BP dosage, inducing the anti-proliferative effects on cancer cells, was fixed at 5 μg/mL, consistent with the dosage used in a previous report [28]. In Figure 1C, the results obtained using Alamar Blue (AbD Serotec, Milano, Italy) assay confirmed that this dosage significantly decreased PC-3 (cancer cells) proliferation after 72 h of exposure to 2D BP flakes compared to the control, i.e., PC-3 cells not seeded with 2D BP flakes, thus confirming the cytotoxic effect of the 2D BP flakes on the PC-3 cells [28].

**Figure 2:**
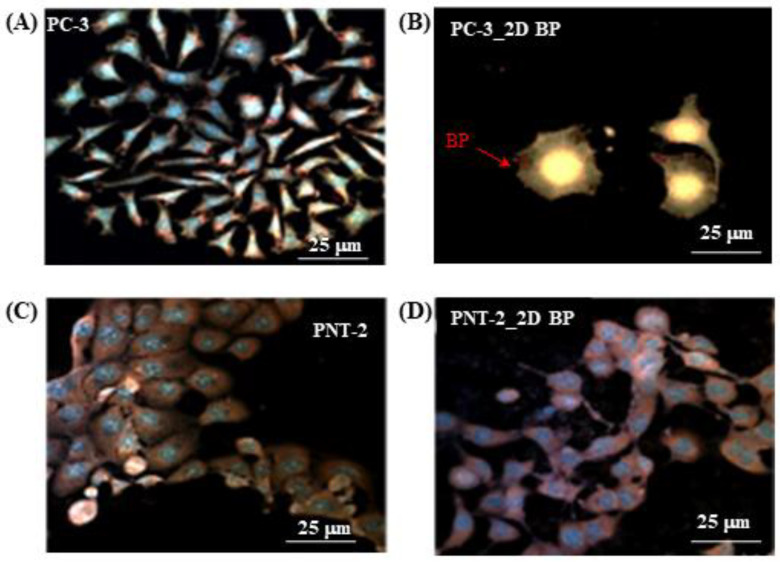
Confocal images on Annexin V-AF647/PI expression by PC-3 and PNT-2 cells in absence ((**A**,**C**), respectively) and in presence ((**B**,**D**), respectively) of 2D BP flakes after 72 h of incubation.

Conversely, the cytotoxic effects observed in PNT-2 after 72 h of exposure to 2D BP were considerably less. Immunofluorescence analysis with rhodamine phalloidin staining, performed after 72 h of cell incubation with and without 2D BP, demonstrated that (1) the PC-3 cells in the absence of the 2D BP flakes reached such a confluence where it was not possible to distinguish cell morphology (Figure 1D); (2) conversely, the presence of the 2D BP flakes induced the disruption of the PC-3 cell body, as is evident in Figure 1E, showing only nuclear and cytoplasmic debris; (3) no morphological changes were observed in the PNT-2 cells (Figure 1G) compared to control sample (Figure 1F). In addition, immunofluorescence analysis performed after 48 h of incubation with the 2D BP flakes (Figure S1 in Supplementary Materials (SM)) gave evidence of PC-3 cells with clear signs of damage: the PC-3 cells lost their classical tapered shape, visible in Figure S1A,B showing the PC-3 cells not exposed to the 2D BP flakes, and appeared as suffering (Figure S1C,D) and apoptotic (Figure S1E) cells or debris (Figure S1F).

To confirm that the incubation of cells with the 2D BP flakes for 72 h at the concentration of 5 μg/mL induced apoptosis in the PC-3 cells, while not causing damage to the healthy ones, we studied the Annexin V-AF647/propidium iodide (PI) expression, widely applied to detect early-phase apoptosis [43].

Annexin V produces a green fluorescence when it binds to the phosphatidylserine (PS) proteins, which are present on the outside of the apoptotic cell membrane. Due to apoptosis or loss of membrane integrity in necrotic cells, PI can enter cells to stain DNA by causing a red fluorescence. When Annexin V and PI are used in combination, apoptosis of cells is indicated by a yellow fluorescence, which is the result of merging the signals from Annexin and PI binding, respectively, with PS (green fluorescence) and DNA (red fluorescence). In Figure 2A, the Annexin V-AF647/PI signal (yellow signal) is not visible, suggesting that, in the absence of the 2D BP flakes, no apoptosis processes affected the PC-3 cells, which also showed considerable spreading. By contrast, the PC-3 cells appeared in the apoptosis phase after 72 h of exposure to the 2D BP flakes; the cells indeed expressed the Annexin V-AF647/PI merged signal along their entire body (Figure 2B). Additionally, darker areas inside the cell membrane can be observed only in Figure 2B, suggesting the presence of 2D BP in the cell body, which is indicative of PC-3 cell permeability to the 2D BP flakes likely internalized in the tumor cells via macropinocytosis, which is a multi-step endocytosis process capable of bulk endocytosis that is upregulated in the tumor cells [44]. Macropinocytosis is indeed described as the inward folding by some of the cell surface ruffles to fuse with the basal membrane, forming vesicular structures called macropinosomes. The macropinosomes are large, uncoated vesicles that greatly vary in size, with their diameters ranging between 0.2 μm and 5 μm [45]. Thus, macropinocytosis has also been implicated in the active cellular uptake of large nanoparticles, including nanosized BP, and nanoparticle aggregates [46,47,48], with sizes ranging from 250 nm to 3–5 μm and without requiring specific targeting moieties on the nanoparticle surface, as in our case. More images of Annexin V-AF647/PI expression by apoptotic PC-3 cells after 72 h of 2D BP exposure are in Figure S2 in SM. Particularly, in Figure S2A, it is possible to observe some PC-3 cells going to apoptosis; in Figure S2B, all PC-3 cells are in an apoptotic condition, corresponding to cells that have lost their normal morphology and have acquired a round shape [49]. Conversely, no apoptosis was visible in the PNT-2 cells treated with 2D BP for 72 h (Figure 2D) by appearing alive and with good cell spreading, as in the control (Figure 2C). These results are in line with our previous study [28], where it was demonstrated that the 2D BP flakes in PC-3 are able to reduce cell vitality by inducing higher ROS production and inactivating the buffer mechanism in terms of GPX-3 (glutathione peroxidase) in basal condition compared to the control (PC-3 without 2D BP). The GPX enzyme family (GPX1–3 and 4) plays a pivotal role in the protection of buffer mechanisms by reducing the hydrogen peroxide’s (H_2_O_2_) and lipid hydroperoxides’ levels.

### 3.2. Label-Free Imaging of the Delivery of the 2D BP Flakes in the Live PC-3 Cells by ODT

**Figure 3:**
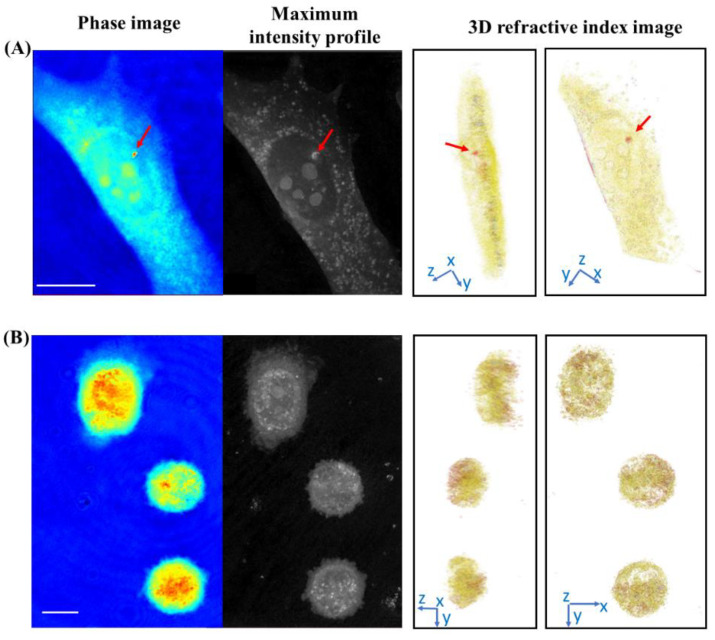
Phase, maximum intensity projection, and 3D RI images of a PC-3 cell after 18 h (**A**) and a group of PC-3 cells after 48 h (**B**) of incubation with 2D BP flakes. In the phase and RI images, the BP clusters are visible as red dots (maximum RI: 1.38) in the cytoplasm marked in yellow (maximum RI: 1.34). Scale bar: 10 μm.

Live imaging of tumor cell internalization of label-free 2D BP flakes was performed by ODT technique. In particular, the 2D BP flakes were added to the cell culture after 24 h of seeding the PC-3 cells on the Petri dish to ensure proper attachment and spreading of the cells on the dish. On the basis of the immunofluorescence investigation, indicating an evident cytotoxic effect on tumor cells after 72 h of exposure to 2D BP, live imaging was performed in the 18–48 h time range after 2D BP administration, i.e., before several cells were killed by the 2D BP action. Figure 3 shows, side by side, the phase, the intensity profile, and the 3D RI images for thePC-3 cells after 18 h (Figure 3A) and 48 h (Figure 3B) of incubation with the 2D BP flakes. The phase, intensity profile, and 3D RI images of the cell after 18 h of the administration of the 2D BP flakes were detailed: the PC-3 cell showed the specific tapered shape, nucleus, and nucleoli of the cell were clearly identifiable, as well as a 2D BP cluster (seen as a red dot with a higher value of RI in the phase and the RI image, indicated by a red arrow). The RI images of the cell observed from different orientations (Movie S1 in the SI) directly confirmed the internalization of the 2D BP. Moreover, at 18 h, the 2D BP cluster was observed to be near the nucleus in the 3D RI image (Figure 3A). Other, smaller-sized 2D BP flakes, not resolved in the ODT images, might also have been present in several places inside the cell, including in the cytoplasm (Figure S3 in the SI). After 48 h of incubation, we observed the presence of a large amount of 2D BP flakes (red dots in Figure 3B) in several places inside the cell (Movie S2 in the SI). In addition, the cells were characterized by a rounded shape and a reduced size with respect to the initial state, which are characteristics of ongoing apoptosis processes [49]. We further performed a detailed morphological characterization of the cells using the collected maximum intensity projections’ (MIP) images at the 36–48 h time range, since the immunofluorescence analysis indicated an onset of apoptosis in that time range.

**Figure 4:**
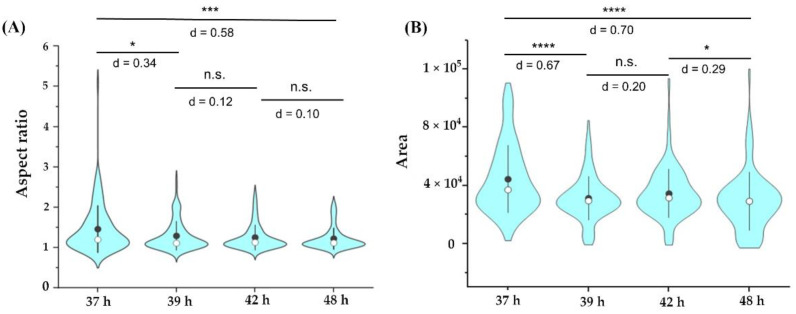
Violin plots showing the distribution of aspect ratio (**A**) and cell area (**B**) at different time points after 2D BP exposure (106 cells considered at each time point). Black dot indicates the mean and the white dot indicates the median of the distribution; * indicates statistically significant differences at the threshold value of *p* < 0.05; n.s.: not a statistically significant difference (* *p* < 0.01; *** *p* < 0.0001; **** *p* < 0.00001). Cohen’s d marked in the figure indicates the effect size for the *t*-test.

Figure 4A,B shows the violin plots for the distribution of the cell aspect ratio and cell area, respectively, at different time points after the administration of the 2D BP flakes. A total of 106 cells for each time point were considered for the distribution. It was observed that, at 37 h, many of the cells had elongated shapes, which became round as time progressed (Figure 4A). Additionally, there was a decrease in the cell area with time (Figure 4B). This indicated that the cells rapidly reached an apoptotic stage within 36–48 h after the 2D BP flakes were administrated.

### 3.3. In Situ Characterization of the Degradation of 2D BP Flakes in the Intracellular Matrix of PC-3 Cells by Raman Spectroscopy

**Figure 5:**
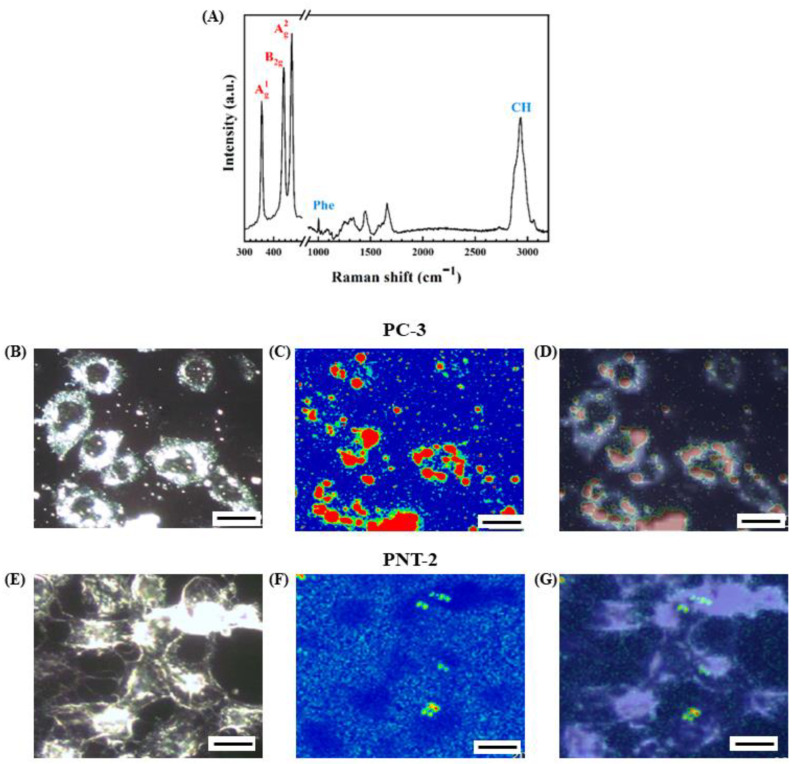
Raman spectral features revealed along the maps and associated to 2D BP flakes, peaks with red indications, and cells’ components, peaks with blue indications, (**A**). Bright-field, Raman map, and bright-field/Raman map overlapped images obtained from PC-3 ((**B**–**D**), respectively) and PNT-2 ((**E**–**G**), respectively) cells after 24 h of seeding with 2D BP. The red contrast in the Raman maps indicates the 2D BP flakes’ presence. Scale bar: 20 μm.

We employed Raman imaging to study the oxidation process of the 2D BP flakes in the intracellular matrix of cancer and healthy cells over a time range from 10 h up to 72 h. Figure 5B,E shows representative optical images for a group of PC-3 and PNT-2 cells, respectively, after 24 h of interaction with 2D BP flakes. The optical images are detailed: the nuclei and the membrane, containing the cytoplasmic region, are clearly identifiable for both groups of cells. Figure 5A presents characteristic spectral features revealed by Raman mapping of the treated cells. Specific bands related to the 2D BP flakes and cellular components included (1) the peaks at 360, 434, and 462 cm^−1^ corresponding to the three vibrational modes of 2D BP, A^1^_g_, B_2g_, and A^2^_g_, respectively [50]; (2) those located between 1000 cm^−1^ and 1654 cm^−1^ ascribed to proteins and lipids of the biological components (such as phenylalanine, Phe, at 1002 cm^−1^); and (3) the pronounced band at 2934 cm^−1^ related to CH-group vibrations of the cells [51]. The Raman maps acquired on the regions of Figure 5B,E are presented in Figure 5C,F by using the intensity of the A^2^_g_ as the contrast parameter so that the red regions indicate the 2D BP distribution. By observing the overlap of the optical and spectral images for PC-3 and PNT-2 cells in Figure 5D,G, respectively, we can see an accumulation of the 2D BP flakes in the tumor cells, while only a few clusters can be identified in the case of healthy cells.

Maps of the 2D BP distribution for PC-3 and PNT-2 cultures were also acquired after 10 h of interaction, observing very few clusters in both the cultures (Figure S4 in SM, Figure S4A–F for PC-3 and PNT-2, respectively). Crucially, after 72 h of interaction, the 2D BP flakes completely invaded the PC-3 cells (see Figure S4G–I in SM), which showed a rounded shape that is characteristic of apoptotic cells [49], while PNT-2 cells maintained a regular cell shape with a few 2D BP clusters around the external membrane (Figure S4L–N in SM). Our observations suggest that, within the first 24 h of exposure, the 2D BP flakes abundantly penetrated the cytoplasm of the PC-3 cells (Figure 5C) but they mostly remained external to the PNT-2 cells, as also seen in the immunofluorescence measurements.

To gain insight into the oxidation pathway of the 2D BP flakes while interacting with tumor cells, we analyzed Raman spectra acquired from the 2D BP cluster inside the cells. Although a previous report [36] essentially used the variation of the intensity related to the 2D BP peaks of the A^1^_g_, B_2g,_ and A^2^_g_ Raman bands to decipher the progress of the oxidation process of single flakes in ambient conditions, in the case of cell cultures, this feature conveys poor and misleading indications. This is because the measured intensity depends strongly on the number of 2D BP flakes accumulated in the sampled cellular location, which is a feature that cannot be controlled experimentally.

**Figure 6:**
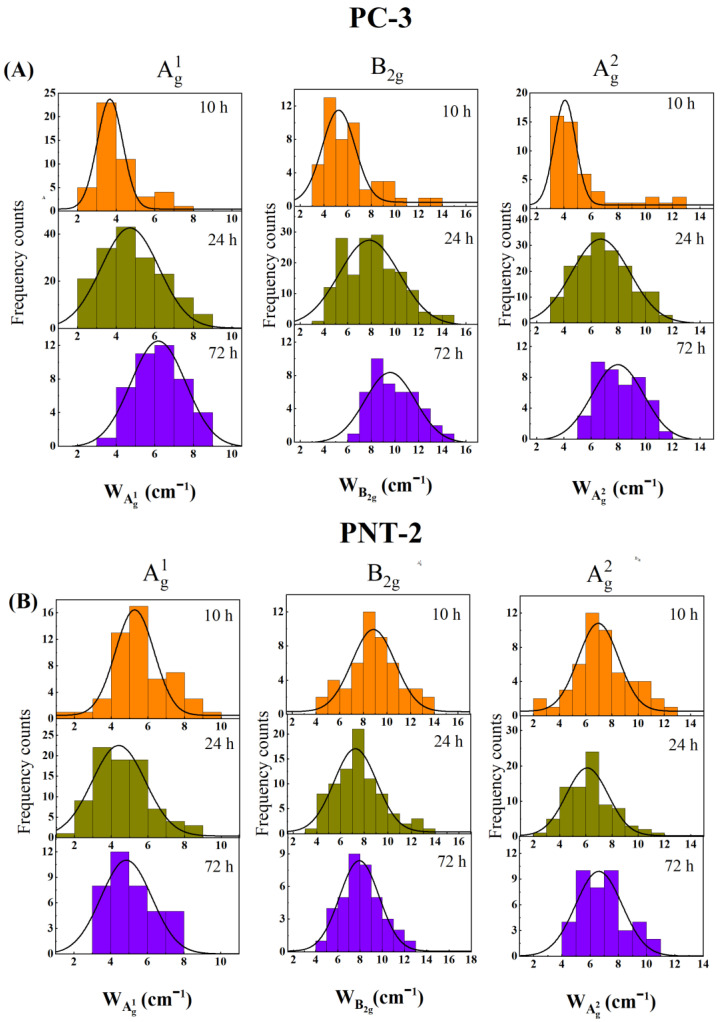
Histogram distributions of the Raman FWHM (W) of A^1^_g_, B_2g,_ and A^2^_g_ vibrational modes of 2D BP inside PC-3 (**A**) and PNT-2 (**B**) cells after 10 h (orange histograms), 24 h (green histograms), and 72 h (violet histograms) of incubation. The superimposed black lines are the results of the Gaussian fit of the data.

On the other hand, the FWHM of the A^1^_g_, B_2g,_ and A^2^_g_ Raman peaks was scarcely influenced by the number of BP flakes accumulated in the analyzed cell location and depended primarily on the crystalline quality and oxidation level of 2D BP [36,52,53]. The broadening of the three Raman peaks as a function of time is, thus, an unambiguous sign of the advanced stages of the degradation of the 2D BP. While most of the studies concentrated on analyzing a single flake over several days to monitor the progression of oxidation, such a method is highly impractical when live cells are involved, which are usually motile and also divide. Moreover, the formation of close aggregates or clusters with gradually increasing size after the cell uptake of 2D BP could completely hide the 2D BP flake under investigation. To overcome these critical issues, we adopted the following methodology: Raman FWHM values (W) of A^1^_g_, B_2g,_ and A_2g_ vibrational modes were extracted from several spectra obtained by mapping 2D BP clusters localized in the PC-3 and PNT-2 cells fixed after different times of exposure. The clusters were identified by using the overlap between optical images and Raman maps, as can be seen in Figure 5D,G, illustrating the representative case of a PC-3 cell and a PNT-2 cell after 24 h of incubation with 2D BP (red regions). In Figure 6A,B, the FWHM distribution of A^1^_g_ (left panel), B_2g_ (central panel), and A^2^_g_ (right panel) is shown for PC-3 and PNT-2, respectively, after 10 h (orange histograms), 24 h (green histograms), and 72 h (violet histograms) of incubation with 2D BP flakes. To exclude any influence of the morphological/structural variations related to different fabrication batches of the 2D BP flakes, for each kind of cellular culture we used 2D BP flakes from the same batch. The superimposed black lines in Figure 6 are Gaussian fits of the data, clearly showing that, in the case of the PC-3 cultures, the FWHM of all the three Raman modes increased with time. This result suggested that the oxidation of the 2D BP flakes was immediately activated as the flakes penetrated inside the tumor cells and completely developed after 10–24 h of incubation, progressively inducing the biological processes leading to the tumor cells’ apoptosis in the successive 36–48 h, as indicated by ODT experiments. In addition, to control potential oxidation effects related to the culture medium, we also mapped and extracted the Raman FWHM of all three vibrational modes of the 2D BP flakes located outside the PC-3 cells after 24 h of incubation. Figure S5 in SM shows minimal changes between the FWHM distribution of A^1^_g_, B_2g,_ and A^2^_g_ for the 2D BP flakes inside and outside the PC-3 cells after 10 h and 24 h of incubation, respectively. This indicates that the changes in the FWHM distribution observed for the 2D BP clusters inside the PC3 cells in the time range of 10–24 h (first and second lines of Figure 6A) were completely due to the intracellular matrix action. Differently, in the PNT-2 cultures, the Raman FWHM of all three vibrational modes remained quite unaltered over the entire observed period (Figure 6B), meaning that the few 2D BP flakes penetrating normal cells were not affected by the intracellular environment.

## 4. Conclusions

The combination of ODT and Raman spectroscopy enables monitoring of the intracellular delivery and the oxidation process of 2D BP flakes in PC-3 cells in situ and in the early stage of 2D BP flakes–cell interaction. While the ODT technique allows us to probe the morphological features and the uptake of 2D BP flakes in living cells without altering their physiological status with the use of any exogenous dye, the Raman imaging enables us to capture the chemical changes of the 2D BP flakes internalized in cancer and healthy cells. Collectively, our results show that 2D BP flakes are internalized by cancer cells very efficiently in less than 24 h of exposure, with some flakes translocating near to the nucleus. The 2D BP flakes oxidize in the presence of the intracellular environment by leading the cells into an apoptotic state gradually. The onset of the apoptotic phase is observed in the time range between 36–48 h. Conversely, the 2D BP flakes incubated with a PNT-2 cellular population remain stable over 3 days of observation, the flakes are scarcely internalized by the cells, and oxidation processes are not activated. We envision that the proposed characterization methodology will be further useful to investigate the dependence of the 2D BP anticancer activity on the flakes’ structural and chemical properties, such as size and surface coatings, in order to achieve a precise and effective tailoring of 2D BP flakes’ properties as anticancer agents. Finally, the application of this approach will be useful for a large variety of cellular studies involving the incorporation and subsequent interaction of therapeutic and diagnostic nanomaterials.

## Data Availability

The data presented in this study are available on request from the corresponding authors.

## Supplementary Materials

The following supporting information can be downloaded at https://www.mdpi.com/article/10.3390/nano12121994/s1. Figure S1: Effect of 2D BP on PC-3 cell morphology (immunofluorescence analysis). Images are representative of PC-3 cells without 2D BP (A,B) and after 48 h of incubation with 2D BP (5 μg/mL) (C–F). The scale bar is 20 μm. The PC-3 cells without 2D BP are characterized by their classic tapered shape (Figure S1A,B), while they appear as suffering (Figure S1C,D) and dying (Figure S1E) cells or debris (Figure S1F) after 48 h of incubation with 2D BP. Figure S2: Confocal images on Annexin V-AF647/PI expression by PC-3 cells after 72 h of 2D BP exposure: some PC-3 cells going to apoptosis (A) and all PC-3 cells in apoptotic phase (B). Scale bar is 50 μm. Figure S3: Phase, intensity profile, and 3D refractive index images of a PC-3 cell after 18 h. Scale bar is 10 μm. Several BP flakes are visible in the cytoplasm. Figure S4: Bright-field image, Raman map, and bright-field/Raman map overlap images obtained from PC-3 (A–C, respectively) and PNT-2 (D–F, respectively) cells after 10 h of seeding with 2D BP (5 μg/mL). Bright-field image, Raman map, and bright-field/Raman map overlap images obtained from PC-3 (G–I, respectively) and PNT-2 (L–N, respectively) cells after 72 h of seeding with 2D BP. The red contrast in the Raman maps indicates the 2D BP presence. The scale bar is 20 μm. Figure S5: Histogram distributions of the Raman FWHM (W) of A^1^_g_, B_2g,_ and A^2^_g_ vibrational modes of 2D BP inside PC-3 cells after 10 h (orange histograms) and outside PC-3 cells after 24 h (green histograms) of incubation. The superimposed black lines are the results of the Gaussian fit of the data. Movie S1: RI images of the 2D BP internalized in PC-3 cell after 18 h of incubation. Movie S2: RI images of the 2D BP internalized in PC-3 cell after 48 h of incubation.
