# Supplementary material for: Label-Free Morpho-Molecular Imaging for Studying the Differential Interaction of Black Phosphorus with Tumor Cells
Source: Nanomaterials (Basel). 2022 Jun 10;12(12):1994. doi: 10.3390/nano12121994 (PMC9227604; doi:10.3390/nano12121994)
Supplement: Supplementary file 1 [file nanomaterials-12-01994-s001.zip › nanomaterials-1734165-supplementary.pdf]

nanomaterials

Label-Free Morpho-Molecular Imaging for Studying the Differential Interaction of Black Phosphorus with Tumor Cells

Valentina Mussi [1,†], Ines Fasolino [2,†], Debadrita Paria [3,†], Sara De Simone [1], Maria Caporali [4], Manuel Serrano-Ruiz [4], Luigi Ambrosio [2], Ishan Barman [3,5,6,*,‡], Maria Grazia Raucci [2,*,‡] and Annalisa Convertino [1,*,‡]

[1] Institute for Microelectronics and Microsystems, National Research Council, 00133 Rome, Italy; valentina.mussi@cnr.it (V.M.); sara.desimone@artov.imm.cnr.it (S.D.S.)
[2] Institute of Polymers, Composites and Biomaterials, National Research Council, 80125 Naples, Italy; ines.fasolino@gmail.com (I.F.); luigi.ambrosio@cnr.it (L.A.)
[3] Department of Mechanical Engineering, Johns Hopkins University, Baltimore, MD 21218, USA; dparia1@jhu.edu (D.P.)
[4] Institute for the Chemistry of Organometallic Compounds, National Research Council, 50019 Sesto Fiorentino, Italy; maria.caporali@iccom.cnr.it (M.C.); manuel.serrano@iccom.cnr.it (M.S.-R.)
[5] Department of Oncology, Johns Hopkins University School of Medicine, Baltimore, MD 21218, USA
[6] Department of Radiology & Radiological Science, Johns Hopkins University School of Medicine, Baltimore, MD 21218, USA
* Correspondence: ibarman@jhu.edu (I.B.); mariagrazia.raucci@cnr.it (M.G.R.); annalisa.convertino@cnr.it (A.C.)
† These authors contributed equally to this work.
‡ These authors contributed equally to this work.

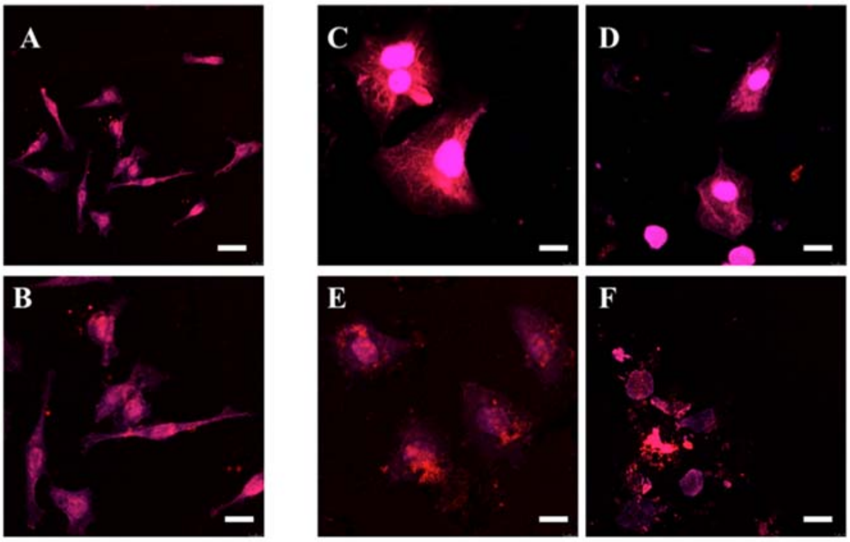

Figure S1. Effect of 2D BP on PC-3 cell morphology (immunofluorescence analysis). Images are representative of PC-3 cells without 2D BP (A and B) and after 48 h of incubation with 2D BP (5 µg/mL) (C, D, E, F). The scale bar is 20 mm. The PC-3 cells without 2D BP are characterized by their classical tapered shape (Figure S1A and S1B), while they appear as suffering (Figure S1C and S1D) and dying (Figure S1E) cells or debris (Figure S1F) after 48 h of incubation with 2D BP.

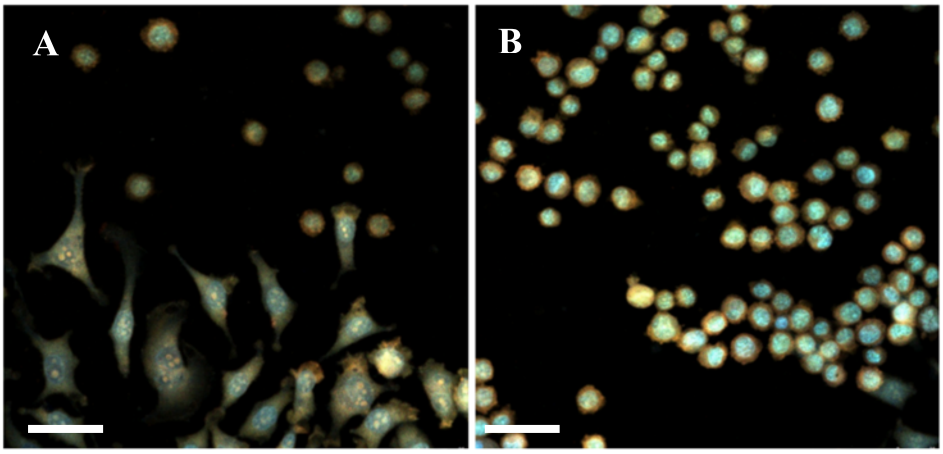

Figure S2. Confocal images on Annexin V-AF647/PI expression by PC-3 cells after 72 h of 2D BP exposure: some PC-3 cells going to apoptosis (A) and all PC-3 cells in apoptotic phase (B). Scale bar 50 µm.

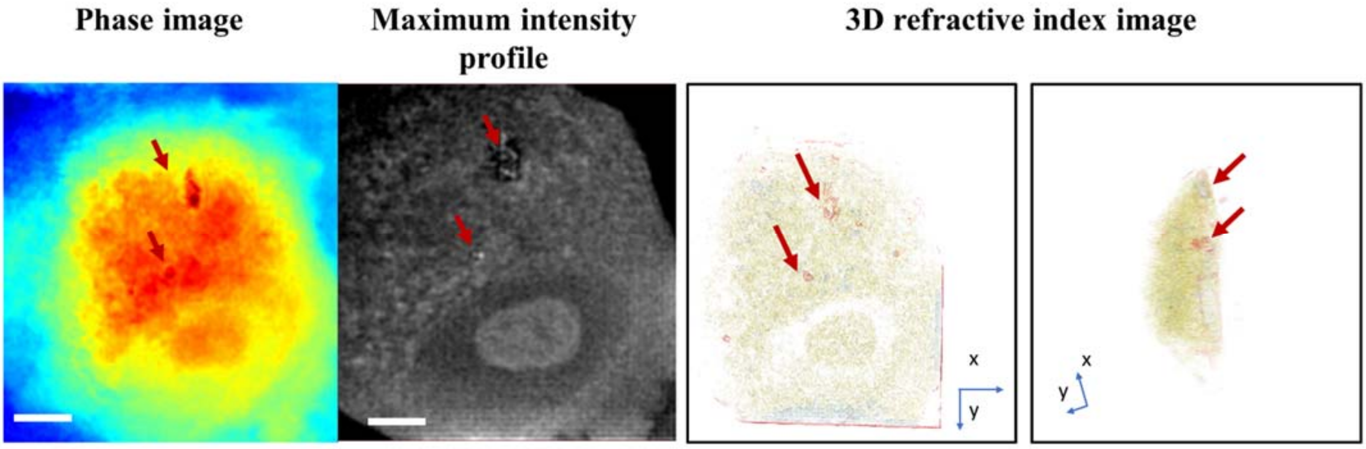

Figure S3. Phase, intensity profile and 3D refractive index images of a PC-3 cell after 18 h. Scale bar 10 µm. Several BP flakes are visible in the cytoplasm.

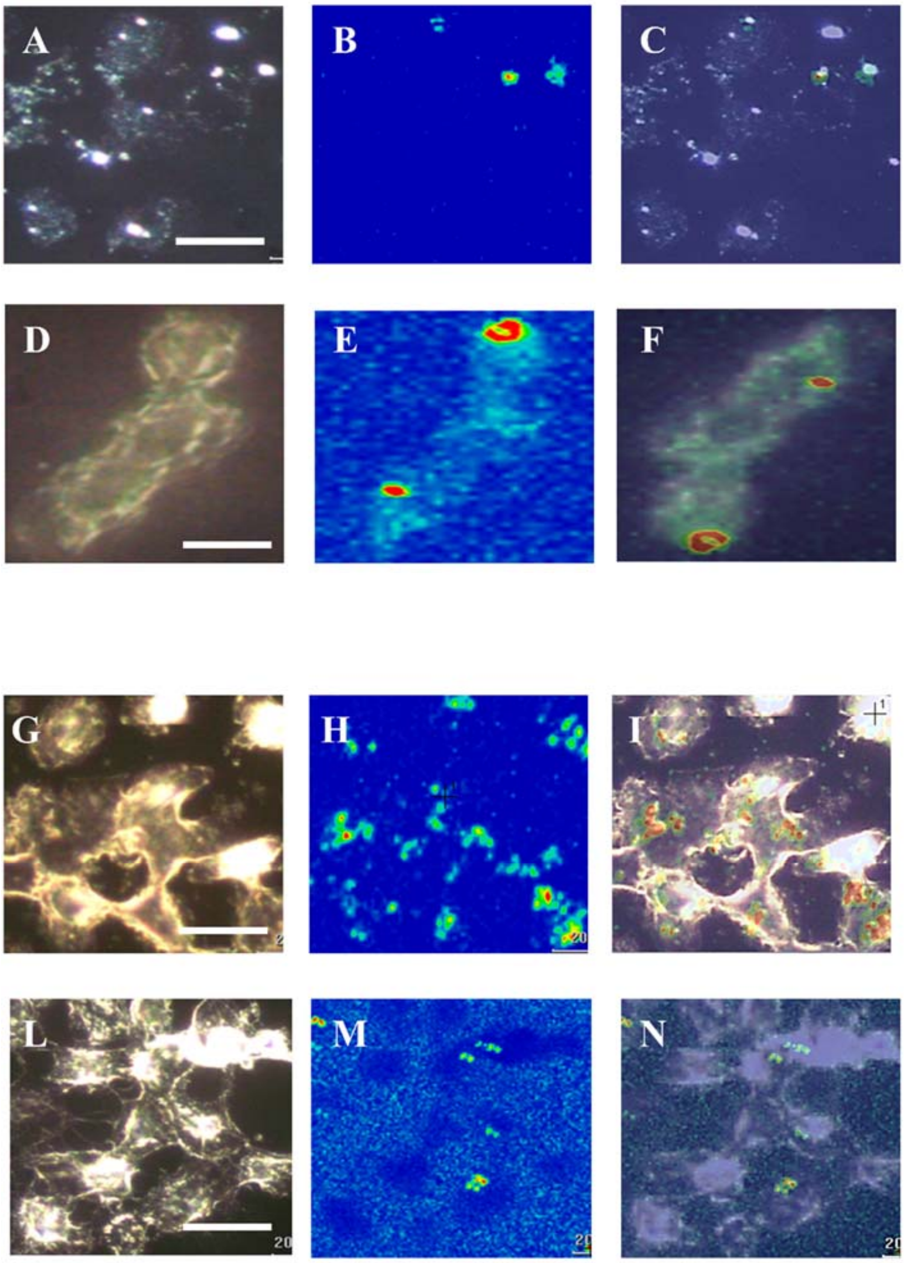

Figure S4. Bright-field image, Raman map and bright-field /Raman map overlap images obtained from PC-3 (A, B, C, respectively) and PNT-2 (D, E and F, respectively) cells after 10 h of seeding with 2D BP (5 µg/mL). Bright-field image, Raman map and bright-field /Raman map overlap images obtained from PC-3 (G, H and I respectively) and PNT-2 (L, M and N, respectively) cells after 72 h of seeding with 2D BP. The red contrast in the Raman maps indicates the 2D BP presence. The scale bar is 20 µm.

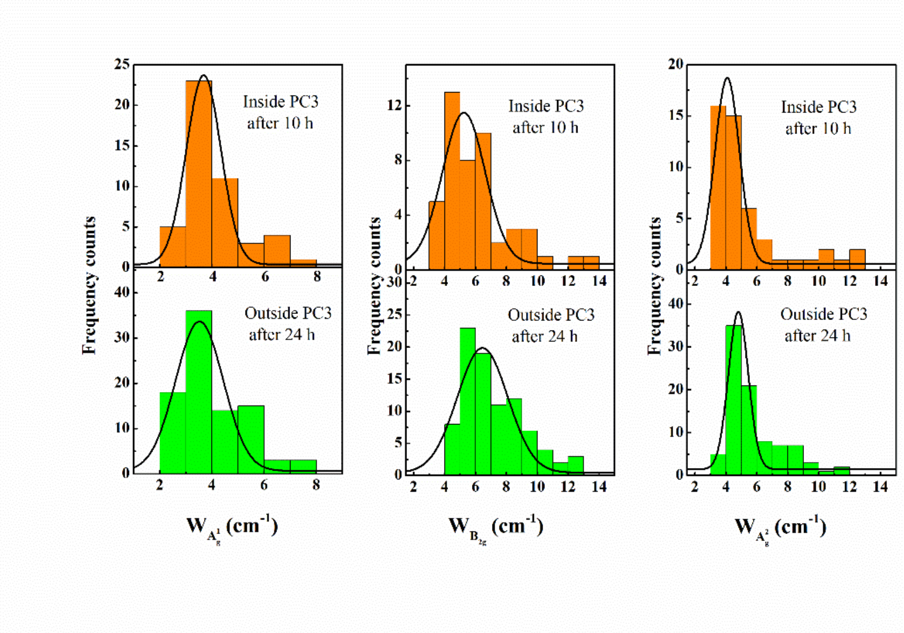

Figure S5. Histogram distributions of the Raman FWHM, *W*, of A^1_g , B_{2g} and A^2_g vibrational modes of 2D BP inside PC-3 cells after 10 h (orange histograms) and outside PC-3 cells after 24 h (green histograms) of incubation. The superimposed black lines are the results of the Gaussian fit of the data.
